# Behavioral Expression of Contextual Fear in Male and Female Rats

**DOI:** 10.3389/fnbeh.2021.671017

**Published:** 2021-06-18

**Authors:** Amanda S. Russo, Ryan G. Parsons

**Affiliations:** Department of Psychology, Stony Brook University, Stony Brook, NY, United States

**Keywords:** context, fear, female, sex differences, freezing, fear-potentiated startle

## Abstract

The study of fear conditioning has led to a better understanding of fear and anxiety-based disorders such as post-traumatic stress disorder (PTSD). Despite the fact many of these disorders are more common in women than in men, the vast majority of work investigating fear conditioning in rodents has been conducted in males. The goal of the work presented here was to better understand how biological sex affects contextual fear conditioning and expression. To this end, rats of both sexes were trained to fear a specific context and fear responses were measured upon re-exposure to the conditioning context. In the first experiment, male and female rats were given context fear conditioning and tested the next day during which freezing behavior was measured. In the second experiment, rats were trained and tested in a similar fashion while fear-potentiated startle and defecation were measured. We found that males showed more freezing behavior than females during a fear expression test. The expression of fear-potentiated startle did not differ between sexes, while males exhibited more defecation during a test in a novel context. These data suggest that the expression of defensive behavior differs between sexes and highlight the importance of using multiple measures of fear when comparing between sexes.

## Introduction

The prevalence of some fear and anxiety-based psychopathologies differs between sexes, including post-traumatic stress disorder (PTSD), which is about twice as common in women as it is in men (Breslau et al., 1998; Kilpatrick et al., 2013). The traumatic event that initiates the dysregulated fear response characteristic of PTSD is readily identifiable and is akin to a Pavlovian fear conditioning procedure with cues present at the time of trauma becoming associated with the traumatic experience (Parsons and Ressler, 2013). One hallmark of PTSD is that fear responses are not restricted to the cues present at the time of trauma, but instead generalize to stimuli not originally associated with trauma (Jovanovic et al., 2012; Kaczkurkin et al., 2017). Much of the ability to restrict fear responses to the appropriate stimuli has to do with the successful recognition of contextual cues (Maren et al., 2013). Thus, studying contextual fear conditioning in rodents might offer some insight into this key aspect of PTSD.

Contextual fear conditioning describes when an organism learns to associate an aversive stimulus with the context in which it was delivered. Contextual fear conditioning has been studied in the laboratory for several decades leading to many advances in both the understanding of fear behavior and its underlying neural systems. A handful of studies have compared contextual fear conditioning between sexes, and the results of these are equivocal. Some studies have found that male rats show higher levels of contextual fear when compared to female rats (Maren et al., 1994; Wiltgen et al., 2001; Chang et al., 2009; Barker and Galea, 2010), others have shown no differences (Kosten et al., 2006; Dachtler et al., 2011; Keiser et al., 2017), and some have reported that females showed more contextual fear than males (Fenton et al., 2016). These discrepancies likely reflect the influence of multiple factors including parametric differences among studies (e.g., Wiltgen et al., 2001). Another factor complicating the comparison of males and females is that there is evidence that the behavioral expression of fear differs between sexes (Dalla et al., 2008; Gruene et al., 2015). If the behavioral expression of fear differs between males and females, then in some cases differences between sexes in fear conditioning might be attributable to differences in behavioral performance, and not necessarily learning.

The approach adopted here was chosen with the hope that it might offer some clarity with respect to sex differences in contextual fear learning. Prior work (Archer, 1975; Blanchard et al., 1991; Dalla et al., 2008; Gruene et al., 2015) indicates that defensive behaviors between sexes in rodents differ in important ways, but less is known about how contextual fear conditioning and expression differ between male and female rodents. Our hypothesis was that if contextual fear conditioning differed between males and females then this difference should be observed on all measures of fear. If instead, differences in freezing behavior were influenced by performance variables, then differences between males and females might be specific to certain measures of fear. To test our hypothesis, male and female rats were exposed to two contextual fear conditioning procedures with identical training and testing parameters. Fear was assessed by measuring freezing behavior, fear-potentiated startle, and conditioned defecation. Fear-potentiated startle and freezing behaviors are two commonly measured defensive behaviors activated by learned fear, and both are thought to be part of the post-encounter defensive mode (Fanselow, 1994). Variability in the expression of these, and other behaviors in rodents is relevant to the variability in response to trauma in humans (Cohen et al., 2003, 2004; Yehuda and LeDoux, 2007). This is especially true for acoustic startle, which is known to be exaggerated in PTSD (Morgan et al., 1995; Grillon and Baas, 2003; Pole et al., 2009). By keeping parameters consistent while assessing multiple measures of fear, we hoped to be able to determine whether males and females show different levels of contextual fear learning that would be observed across all measures, or whether any potential differences were specific to certain fear responses. Our findings indicate that differences in contextual fear were observed when measuring freezing behavior, with males showing higher freezing levels during testing in the conditioning context. However, levels of both fear-potentiated startle and defecation did not differ between sexes when rats were tested in the conditioning context. Our data indicate that sex differences in contextual fear are not observed broadly across all measures, suggesting that the behavioral expression of contextual fear, but not learning *per se*, differs between male and female rats.

## Method

### Subjects

Thirty-three, adult, male Sprague–Dawley rats (300–325 g upon arrival) and 35, adult, female Sprague–Dawley rats (200–225 g upon arrival), obtained from Charles River Laboratories (Raleigh, NC, USA) served as subjects (approximately 8–10 weeks of age). The rats were housed in pairs in plastic boxes, with food and water freely available, on a 12 h light/dark cycle (lights on at 7 am). All experiments took place during the light portion of the light/dark cycle. All procedures were approved by the Stony Brook University Institutional Animal Care and Use Committee and were in accordance with the National Institutes of Health guidelines for the care and use of laboratory animals.

## Behavioral Apparatus

### Experiment 1: Freezing

The apparatus for all experiments has been described in detail elsewhere (Russo and Parsons, 2017). Experiment 1 took place in conditioning chambers (Clever Systems Inc., Reston, VA, USA) located within sound-attenuating isolation boxes. The conditioning chambers contained shock grid floors and stainless steel and Plexiglas walls, 28-V, incandescent, house light bulbs, and were wiped down with 5% acetic acid. Overhead cameras recorded behavioral sessions and the video signal from each chamber fed into a software program (FreezeScan 2.00) which automatically scored freezing behavior based on pixel change. Parameters for scoring were chosen such that the computer-scored freezing behavior closely matched hand-scored behavior by a trained observer, and the motion parameters were set as follows (noise filtering radius = 1, interframe motion < 100 pixels, Freeze *N* = 24, Freeze *M* = 22, Move *N* = 10, Move *M* = 8).

### Experiments 2 and 3: Fear-Potentiated Startle and No-Shock Controls

Experiments 2 and 3 took place in sound-attenuating cabinets (Startle Monitor II, Kinder Scientific, Poway, CA, USA). Fear conditioning and a context fear test took place in Context A, where rats were placed in cages made of Plexiglass and a stainless-steel shock-grid floor, the house lights in the cabinets were turned on, the ceiling lights in the lab were turned off, and the cages were wiped down with 5% ammonium hydroxide. Baseline startle response and a second context fear test took place in Context B, where rats were placed in restrainers made with a stainless-steel rod cover and a plastic floor, the house lights in the cabinets were turned off, the ceiling lights in the lab were turned on, and the cages were wiped down with 70% EtOH.

Both the shock cages and the restrainers sat on top of load cell sensing platforms inside the cabinets. Startle amplitude was reported in Newton (N) through a single-pulse calibrator interfaced to a PC. Startle amplitude was defined as the peak N that occurred during the 500 ms following the onset of a white noise burst. Startle responses were elicited by 50 ms, 95 dB, white noise bursts which were delivered through speakers mounted on the ceilings of the cabinets. Shocks were delivered through a grid floor.

## Behavioral Procedures

### Experiment 1: Freezing

Rats were handled for 5 min per day for 7 days before behavioral procedures began. The first 4 days of handing occurred in the colony room. For the final 3 days, rats were carted into the laboratory and handled. On the first day of the experiment, rats (*n* = 14 of each sex) were placed into the conditioning chambers where they were exposed to three, 1 mA, 1 s foot shocks (20 s ITI) following a 4 min baseline period. Rats were returned to their home cages 2 min following the last shock. The following day, all rats were placed back into the conditioning chamber for a 10 min context test. Approximately half of the male rats (*n* = 8) and half of the female rats (*n* = 8) were run by a female experimenter, while the remaining male rats (*n* = 6) and female rats (*n* = 6) were run by a male experimenter.

### Experiment 2: Fear-Potentiated Startle

The same handling procedure was used as described above. On the first 2 days of the experiment, baseline startle was measured by placing rats (*n* = 14 females, *n* = 11 males) into startle chambers (Context B) and exposing them to 30, 95 dB, 50 ms, white noise bursts (30 s ITI) following a 5 min baseline period. The following day, rats were placed into Context A and were exposed to three, 1 mA, 1 s foot shocks (20 s ITI) following a 4 min baseline period. Rats were returned to their home cages 2 min following the last shock. Three male rats were excluded from the analysis due to a technical malfunction on the conditioning day. The next day, rats were tested for fear-potentiated startle in Context A for 10 min. During this session, rats were exposed to 20, 95 dB, 50 ms, white noise bursts (30 s ITI) following a 30 s stimulus-free period. On the last day of the experiment, rats were tested for fear-potentiated startle in Context B with stimuli identical to those presented during the Context A test. The number of fecal boli produced by each rat was recorded after each testing session. Approximately half of the male rats (*n* = 5) were run by a male experimenter, while the remaining male rats (*n* = 6) and all of the female rats (*n* = 14) were run by a female experimenter.

### Experiment 3: No-Shock Controls

The same handling procedure was used as described above. Baseline startle was measured by placing rats (*n* = 8 males, *n* = 7 females) into startle chambers on consecutive days (Context B) and exposing them to 30, 95 dB, 50 ms, white noise bursts (30 s ITI) following a 5 min baseline period. The following day, rats were placed into Context A for 7 min, however, no shock was delivered. As in Experiment 2, the next day rats were tested in Context A for 10 min, and 24 h later were given a test in context B. One female rat was excluded from the analysis due to a technical malfunction on the conditioning day. Acoustic startle and defecation were measured during both of the test sessions, as described in Experiment 2. All of the male rats were run by a male experimenter, while all of the female rats were fun by a female experimenter.

## Data Analysis

### Experiment 1: Freezing

Average time spent freezing during the baseline period and the post-shock period of the fear conditioning session was averaged for each animal. Likewise, the average time spent freezing during the 10 min context test was computed for all rats. Shock reactivity and post shock activity bursts were analyzed by computing motion (defined by the number of pixel changes/frame) during the 5 s before shock, the 1 s during shock, and the 5 s after shock. Independent samples *t*-tests (one-tailed) were used to compare freezing between groups.

### Experiment 2 and 3: Fear-Potentiated Startle and No-Shock Controls

Baseline startle values were calculated by taking the average of startle responses across the 2 days of baseline startle testing. Context fear-potentiated startle was calculated by first subtracting the average baseline startle response from the startle response during the test sessions to produce a difference score, and then dividing the difference score by the baseline startle mean and multiplying by 100 to produce a fear-potentiated startle percentage. Independent samples *t*-tests (one-tailed) were used to compare between groups, and repeated measures ANOVAs were used for within-and between-subject comparisons. Mann–Whitney *U* tests were used to compare males and females in fecal boli counts. Results were considered significant when *p* < 0.05 for all statistical tests. For each *t*-test reported, Cohen’s *d* is also reported, with 0.2, 0.5, and 0.8 being considered small, medium, and large effect sizes, respectively.

## Results

**Figure 1:**
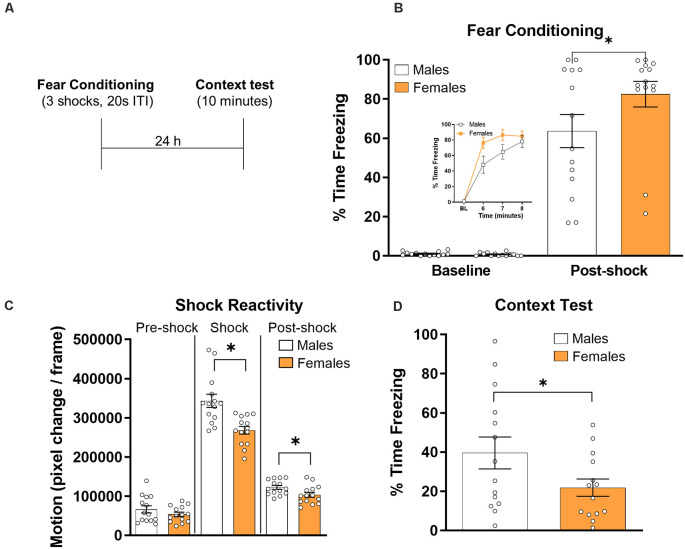
Male (*N* = 14) and female (*N* = 14) were given contextual fear conditioning and freezing behavior was assessed during a 10 min test session the next day (panel** A** depicts the timeline of the experiment). **(B)** Freezing behavior during the baseline and post-shock periods during the fear conditioning session. The inset graph shows average baseline freezing and minute-by-minute freezing during the last 3 min of the conditioning session. **(C)** Shock reactivity and post shock activity burst as measured by the average number of pixel changes per frame for male and female rats during the 5 s before the shock (left panel), during the 1 s duration of the shock (middle panel), and during the 5 s after the shock (right panel). Freezing behavior during the context test session in male and female rats **(D)**. For all graphs, symbols reflect individual subject values and error bars reflect the standard error of the mean. *p < 0.05.

All rats were given a fear conditioning session followed 24 h later by a test session in the training context (Figure 1A). We first compared freezing levels across groups during the fear conditioning session by averaging freezing levels during the baseline and post-shock periods for all rats (Figure 1B). A *t*-test revealed that baseline freezing did not differ between groups (*t*_(26)_ = 1.02, *p* > 0.05, *d* = 0.38). A similar analysis on the data from the post-shock period revealed a significant effect of group (*t*_(26)_ = −1.76, *p* < 0.05, *d* = 0.67), with female rats showing higher freezing levels overall.

Shock reactivity was analyzed for male and female rats (Figure 1C). Motion levels were similar for males and females during the 5 s prior to the shock (*t*_(26)_ = −1.18, *p* > 0.05, *d* = 0.45), but males showed significantly more motion during the 1 s shock (*t*_(26)_ = −3.85, *p* < 0.001, *d* = 1.46) and during the 5 s after the shock (*t*_(26)_ = −2.37, *p* < 0.05, *d* = 0.90).

For the testing data (Figure 1D), freezing levels were averaged across the 10 min session. A *t*-test on these data showed a significant difference between groups (*t*_(26)_ = 1.91, *p* < 0.05, *d* = 0.72), with males showing higher freezing levels during the test session. Because males showed higher reactivity to shock during conditioning and higher levels of freezing during the context test, we examined whether or there was a relationship between the two measures. We computed correlation coefficients using Pearson’s r in both males and females. There was no significant correlation between shock reactivity and freezing during the context test in either males (*r* = −0.20, *p* > 0.05) or females (*r* = −0.20, *p* > 0.05), suggesting that differences in shock reactivity were not driving the differences in freezing behavior during the test session.

**Figure 2:**
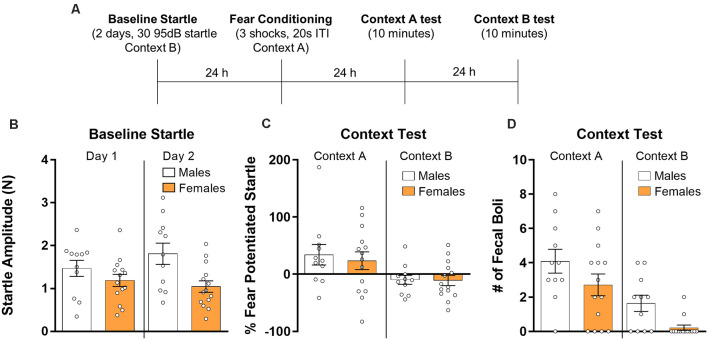
Male (*n* = 11) and female (*n* = 14) rats were given baseline startle tests on consecutive days and the next day they were exposed to contextual fear conditioning. Rats were then exposed to the training context (Context A) and the next day re-exposed to the startle context (Context B) for 10 min during which fear-potentiated startle was assessed (panel **A** depicts the timeline of the experiment). **(B)** Average baseline startle amplitude in males and females during both days of startle testing. **(C)** Fear potentiated startle in the training context (Context A, right panel) and during a test in the startle chamber (Context B, left panel). **(D)** The number of fecal boli in males and females during the respective test sessions.

Next, we analyzed the data from rats given contextual fear conditioning and which were subsequently tested in the conditioning context and in a context in which shock was not delivered (Figure 2A). Fear-potentiated startle was assessed on both test days. We first analyzed baseline startle responses (Figure 2B) using a repeated measures ANOVA with session as a within-subjects factor and sex as a between-subjects factor. Results from this analysis showed that there was no effect of the session (*F*_(1,23)_ = 1.00, *p* > 0.05), indicating that startle responses did not change across the 2 days of testing. There was a significant effect of sex (*F*_(1,23)_ = 5.30, *p* < 0.05) with males having higher amplitude startle responses, and a significant session by sex interaction (*F*_(1,23)_ = 5.87, *p* < 0.05) owing to a further divergence in startle responses between sexes on day 2. Next, we analyzed testing data (Figure 2C) using a repeated measures ANOVA with context as a within-subjects factor and sex as a between-subjects factor. Results from this analysis revealed a significant effect of context (*F*_(1,23)_ = 9.68, *p* < 0.01) driven by a greater fear-potentiated startle in the context in which the animals were shocked. There was no context by sex interaction (*F*_(1,23)_ = 0.13, *p* > 0.05) and no main effect of sex (*F*_(1,23)_ = 0.18, *p* > 0.05). We also used *t*-tests to individually compare males and females for both test sessions. Results from these tests showed no differences between sexes for the test session in Context A (*t*_(23)_ = 0.434, *p* > 0.05, *d* = 0.18) and Context B (*t*_(23)_ = 0.11, *p* > 0.05, *d* = 0.04). Finally, we compared the number of fecal boli (Figure 2D) collected during both test sessions using a repeated measures ANOVA. Results showed a significant effect of context with a higher number of fecal boli during the test in Context A (*F*_(1,23)_ = 23.46, *p* < 0.001). There was no interaction between sex and context (*F*_(1,23)_ = 0.002, *p* > 0.05), but there was a significant effect of sex (*F*_(1,23)_ = 6.89, *p* < 0.05). Mann–Whitney *U* tests were used to compare males and females in fecal boli production in both test sessions. There was no significant difference between males and females in the number of fecal boli during the test in Context A (*U* = 53, *p* > 0.05, *d* = 0.59), however, males showed significantly more fecal boli than females during the test in Context B (*U* = 35, *p* < 0.01, *d* = 1.27).

**Figure 3:**
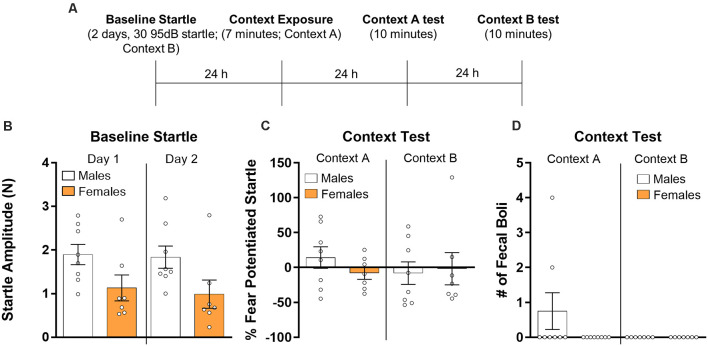
Male (*n* = 8) and female (*n* = 7) rats were given baseline startle tests on consecutive days and the next day they were exposed to a fear conditioning chamber with no shock presented. Rats were then exposed to the training context (Context A) and the next day re-exposed to the startle context (Context B) for 10 min during which fear-potentiated startle was assessed (panel **A** depicts the timeline of the experiment). **(B)** Average baseline startle amplitude in males and females during both days of startle testing. **(C)** Fear potentiated startle in the training context (Context A, right panel) and during a test in the startle chamber (Context B, left panel). **(D)** The number of fecal boli in males and females during the respective test sessions.

Finally, we analyzed the data from rats which were treated identically to those in Experiment 2 but were not administered shock on the conditioning day (Figure 3A). First, we used a repeated measures ANOVA to compare baseline startle responses (Figure 3B) across the 2 days of startle testing. There was no effect of session (*F*_(1,13)_ = 0.76, *p* > 0.05) and the session by sex interaction was also not significant (*F*_(1,13)_ = 0.13, *p* > 0.05). There was a significant main effect of sex (*F*_(1,13)_ = 4.72, *p* < 0.05) with male rats showing higher startle values overall, consistent with our observation in Experiment 2. For the test sessions (Figure 3C), a repeated measures ANOVA with context and sex as factors showed no effect of context (*F*_(1,13)_ = 0.35, *p* > 0.05), no sex by context interaction (*F*_(1,13)_ = 1.12, *p* > 0.05), and no effect of sex (*F*_(1,13)_ = 0.17, *p* > 0.05). We also used *t*-tests to individually compare males and females for both test sessions. Results from these tests showed no differences between sexes for the test session in Context A (*t*_(13)_ = 1.21, *p* > 0.05, *d* = 0.63) and Context B (*t*_(13)_ = −0.23, *p* > 0.05, *d* = 0.12). Finally, we analyzed the fecal boli data (Figure 3D) using a repeated measures ANOVA. We found no effect of context (*F*_(1,13)_ = 1.76, *p* > 0.05), no interaction (*F*_(1,13)_ = 1.76, *p* > 0.05), and no effect of sex (*F*_(1,13)_ = 1.76, *p* > 0.05). Because females produced 0 fecal boli in Context A, and both males and females produced 0 fecal boli in context B, Cohen’s d is not reported for these comparisons. Mann–Whitney *U* tests were used to compare males and females in fecal boli production in both test sessions. There were no significant differences between males and females in fecal boli in Context A (*U* = 21, *p* > 0.05) or Context B (*U* = 28, *p* > 0.05).

## Discussion

In this set of experiments male and female rats were given contextual fear conditioning and we measured different fear responses when rats were re-exposed to the context in which shock was delivered. Our results show that males exhibited higher levels of freezing compared to females when they were returned to the conditioning chamber a day following contextual fear conditioning. This was the case even though freezing levels after shock administration during conditioning were higher in females. When fear-potentiated startle or defecation was measured, males and females did not differ in their levels of contextual fear. Prior work from our lab has also uncovered sex differences in cued fear extinction that were specific to certain measures of fear (Voulo and Parsons, 2017, 2019). The results from those studies are complicated by the fact that the parameters used to induce cued fear differed in the experiments comparing freezing to fear-potentiated startle. Here, we were able to avoid this potential complication by keeping the training and testing parameters identical across experiments. The fact that sex differences in contextual fear are not consistently observed across all measures of fear suggests that the difference in contextual fear seen between males and females when freezing is measured may reflect an effect of behavioral performance, and not of differences in learning.

Although our results are consistent with a behavioral performance interpretation, several alternative explanations and factors that may be affecting our results should be discussed. First, it is possible that the relatively mild training parameters used in the current study resulted in a floor effect in the fear-potentiated startle experiment, obscuring a potential difference between sexes. Some prior studies (McNish et al., 1997) have trained rats with stronger conditioning parameters, and it would be worthwhile to compare males and females under such conditions. Another important consideration is whether or not our results were influenced by presenting the rats with loud startle stimuli, which can serve as an unconditioned stimulus capable of supporting contextual fear conditioning (Cranney, 1987). If so, it is possible that the startle stimulus served as UCS and that the increased startle we observed when rats were trained might reflect generalized fear from having received startle stimuli in Context B. However, we think this is unlikely given that prior studies have shown that low intensity (90–100 dB) startle stimuli are less able to support contextual fear (Cranney, 1987; Perusini et al., 2016) and that our data showed that levels of potentiated startle in un-shocked controls did not differ in Context A compared to Context B, suggesting that enhanced startle in A is not simply a result of having received startle trials in B. Finally, while we kept the key conditioning parameters consistent across experiments, it is possible that differences in the apparatus cues might have affected our results. Namely, the size of the chambers in which freezing or fear-potentiated startle was measured were different, and prior work (Rosen et al., 2008) has shown that levels of freezing behavior can be influenced by the size of the testing chambers.

Several prior studies have compared males and female rodents’ performance in contextual fear conditioning and collectively the results are ambivalent. Some studies have reported males showing stronger context fear conditioning (Maren et al., 1994; Wiltgen et al., 2001; Chang et al., 2009; Gresack et al., 2009; Barker and Galea, 2010; Mizuno et al., 2012; Colon et al., 2018; Colon and Poulos, 2020) whereas others showed equivalent levels of contextual fear in males and females (Kosten et al., 2006; Dachtler et al., 2011; Keiser et al., 2017), and some studies have even reported stronger context fear conditioning in females (Fenton et al., 2016; Blume et al., 2017; Zambetti et al., 2019). Nearly all of these prior studies have used freezing behavior to assess learning, meaning that the discrepant results are not simply because these studies used different measures of fear. It is likely that some combination of parametric inconsistencies and/or differences across studies in species or strain can account for the discordant findings, as these variables are known to influence whether sex differences in contextual fear are observed (Pryce et al., 1999; Wiltgen et al., 2001). Our results cannot be explained by parametric differences because the conditioning and testing parameters were identical across experiments.

For the prior studies that have reported higher levels of contextual fear in males than in females, the possibility that this difference reflects an effect of behavioral performance has typically been addressed by measuring cued fear in the same animals (e.g., Maren et al., 1994). The reasoning follows that if cued fear does not differ between sexes, but contextual fear does, then the deficit in contextual fear is likely one of learning and not of behavioral performance. However, it is possible that the differential outcomes seen in prior studies comparing males and females on cued and contextual fear reflect a “ceiling effect” in performance to the discrete cues and that this masks a potential parallel deficit in cued fear in female rodents. In fact, one prior study reported deficits in cued and contextual fear in some rat strains (Pryce et al., 1999). The extent to which these prior findings can be reconciled by whether the behavioral performance was at ceiling is unclear, however, one approach to address this issue would be to vary the intensity of the conditioning session and determine if cued fear deficits are observed in females when performance is sub-asymptotic. This basic approach was taken by Maren et al. (1994) and their results showed a sex difference in contextual fear when rats were trained with a single trial, but not with three trials. In the same rats, freezing levels to a discrete cue were not different regardless of the number of trials. This would seem to argue against a performance effect interpretation, however levels of freezing to the discrete cue in animals trained with a single trial were very low, raising the possibility of a floor effect. In addition to the ceiling effect issue, another important consideration is whether or not prior studies of cued fear, which by and large only measured freezing behavior, might have revealed a different pattern of findings had other measures of fear been taken. A prime example is the recent characterization of “darting” during cued fear, a behavior that is predominant in females (Gruene et al., 2015).

One limitation of the current study is that the estrous cycle phase was not accounted for in the female animals. Our decision to not assess the estrous phase was motivated by a desire to equate handling conditions between sexes and the fact that our prior work with fear-potentiated startle showed that the estrous phase did not affect the expression or extinction of cued fear (Voulo and Parsons, 2017). Some prior studies have reported differences in contextual fear across stages of the estrous cycle (Markus and Zecevic, 1997; Lynch et al., 2013), although this is an inconsistent finding as others have reported lower levels of contextual fear in females regardless of the estrous phase (Chang et al., 2009). Some other studies (Gresack et al., 2009; Fenton et al., 2016) have found sex differences in contextual fear that are not directly attributable to the estrous phase, and our results are perhaps most readily compared to these reports. While we cannot rule out the possibility that the estrous phase affected our findings, if this were the case, we would have expected greater variability in females than in males, which was not consistently observed in any of the experiments.

The primary goal for this study was to determine if sex differences were present in contextual fear when multiple measures of fear were taken. However, for the experiment in which we assessed fear-potentiated startle, rats were also tested in a context in which shock was not presented, making it akin to a test of contextual discrimination. A number of recent studies (Lynch et al., 2013; Keiser et al., 2017; Asok et al., 2019) have reported that female rodents show a deficit in contextual fear discrimination, where they exhibit higher levels of fear in a novel context compared to males. While our results indicate similar levels of discrimination between sexes, they are not necessarily inconsistent with prior work. First, in two of the prior studies (Lynch et al., 2013; Asok et al., 2019) the deficit in discrimination was only seen when the tests occurred several days or more after training. In our study, testing occurred on consecutive days 1 day after training. Second, one of the studies (Asok et al., 2019) showed a test order effect such that the deficit in discrimination in females was observed when they were first tested in the novel context, but not if they were tested first in the training context. All rats in our study were first tested in the training context, making it likely that the testing order favored discrimination. Another factor supporting discrimination in this experiment was the fact that other than background noise levels, the novel chamber did not share any features with the training chamber. Prior work (Keiser et al., 2017) indicates that rats of both sexes can readily discriminate dissimilar contexts, but that females lose the ability to discriminate when the novel context shares some features with the training context. Finally, the primary motivation behind including a test in a novel chamber in the fear-potentiated startle experiment was not to test for discrimination, but to rule out the possibility that the increase in startle when the rats were tested in the training chamber was simply sensitization of the startle reflex by prior shock exposure, a phenomenon known to occur under certain circumstances (Davis, 1989; Hitchcock et al., 1989; Gewirtz et al., 1998). The fact that startle amplitudes were lower in the novel context than in the conditioning context indicates that the conditioning session did not lead to a long-term sensitization of the startle reflex.

Although we found that males showed higher levels of freezing during the test session, females showed higher levels of freezing during the period after shock during the conditioning session. We examined this further first by assessing freezing levels in each of the 3 min following shock. This showed that the difference between sexes was driven largely by lower freezing in males during the first 2 min, with freezing levels becoming similar by the final minute. Next, we examined activity levels around the time of shock. This analysis revealed that males exhibited higher levels of activity both during the shock period and in the 5 s aftershock. This result is somewhat surprising given that several prior studies have not detected sex differences in shock reactivity (Wiltgen et al., 2001; Greiner et al., 2019; Hoffman et al., 2020) and one showing the opposite pattern as reported here (Gruene et al., 2015). Nonetheless, this suggests that the difference in freezing during the post-shock period can be explained, at least partially, by the fact that males react more to the shock and show a more pronounced post-shock activity burst. Importantly, levels of freezing during the context test did not appear to be driven by differences in shock reactivity as there was no significant correlation between the two measures.

The main finding we report here is that males show higher levels of contextual fear when freezing is measured, but not when fear-potentiated startle or defecation is used to assess fear. Importantly, these results cannot be explained by parametric differences as key parameters were equated across experiments. Our results suggest that deficits in contextual fear in female rats may reflect differences in behavioral performance, and not learning. This suggestion is supported by other studies indicating that the expression of defensive behavior in rodents differs in male and female rodents (Dalla et al., 2008; Gruene et al., 2015; Shansky, 2018). This factor needs to be carefully considered when comparing across sexes in studies of learned fear.

## Data Availability Statement

The raw data supporting the conclusions of this article will be made available by the authors, without undue reservation.

## Ethics Statement

The animal study was reviewed and approved by Stony Brook Institutional Animal Care and Use Committee.

## Author Contributions

AR and RP designed the experiments, collected and analyzed the data, and wrote the manuscript. All authors contributed to the article and approved the submitted version.

## Conflict of Interest

The authors declare that the research was conducted in the absence of any commercial or financial relationships that could be construed as a potential conflict of interest.
